# Use of Coercion or Persuasion in Prehospital Treatment in the Region of Southern Denmark: A Retrospective Study

**DOI:** 10.1111/aas.70317

**Published:** 2026-07-31

**Authors:** Sif Højmark Vobbe, Henriette Bruun, Anne Craveiro Brøchner, Annmarie T. Lassen, Tim Alex Lindskou, Torben Anders Kløjgård, Signe Wegmann Düring, Søren Mikkelsen

**Affiliations:** ^1^ Prehospital Research Unit, Prehospital EMS Region of Southern Denmark Odense Denmark; ^2^ Mental Health Services in the Region of Southern Denmark Middelfart Denmark; ^3^ Forensic Mental Health Research Unit Middelfart (RFM), Department of Regional Health Research University of Southern Denmark Middelfart Denmark; ^4^ Department of Regional Health Research University of Southern Denmark Odense Denmark; ^5^ Department of Anaesthesiology and Intensive Care Kolding University Hospital Kolding Denmark; ^6^ Emergency Medicine Research Unit University of Southern Denmark and Odense University Hospital Odense Denmark; ^7^ Centre for Prehospital and Emergency Research at Danish Centre for Health Services Research Aalborg University Hospital Aalborg Denmark; ^8^ Department of Clinical Medicine Aalborg University Aalborg Denmark; ^9^ Psychiatric Research Unit Psychiatry Region Zealand Slagelse Denmark; ^10^ Department of Clinical Medicine University of Copenhagen Copenhagen Denmark

**Keywords:** ambulances, coercion, emergency medical services, prehospital, psychiatry

## Abstract

**Background:**

The use of coercion or persuasion raises ethical challenges but may be justified when prehospital patients require immediate treatment and are unable to consent. This study aims to describe the characteristics of coerced or persuaded adult pre‐hospital patients in the Region of Southern Denmark.

**Methods:**

A descriptive analysis of ambulance contacts in the Region of Southern Denmark from January through June 2022. We identified all cases meeting the predefined inclusion criteria for coercion or persuasion, with or without police involvement. We classified cases into four categories: coercion (forced admission with police involvement), necessity or urgent need for treatment, persuasion with police involvement, and persuasion without police involvement. Additionally, we describe the study population's substance use, instances of self‐harm, and the overall police involvement in managing these patients.

**Results:**

Within 39,021 prehospital patient contacts, 6743 cases indicated that coercion or persuasion could have occurred. In 416 cases (1.1% of all prehospital contacts), coercion or persuasion was confirmed. Of these, 65 patients were forcibly admitted to the hospital. Forty‐six patients received non‐patient‐sanctioned treatment justified by necessity or an urgent need. Fifty patients were persuaded with police involvement, and 255 were persuaded through paternalistic means without police involvement. The median age of the coerced patients was 56 years; 54.8% being men. 26.9% of patients subjected to coercion were under the influence of substances. 17.8% of all coerced patients exhibited self‐harming behaviours, such as attempting suicide, self‐harm, or suicidal thoughts. The police were involved in 31.5% of cases of coercion.

**Conclusion:**

Coercive measures or persuasion to consent were identified in a small proportion of prehospital contacts. When they occur, the most common form is persuasion without police involvement, but other forms of coercion are also observed. The study emphasises the need for greater attention to this issue to ensure the appropriate use of coercion and persuasion during emergencies.

**Editorial Comment:**

This study presents experience from one region of Denmark with ambulance missions where the patient was uncooperative but needed acute medical care. Different approaches were commonly implemented in this circumstance, to try to minimise harm related to administering urgent or emergent care.

**Abbreviation**
EMS: emergency medical service

## 1 Background

Ambulance personnel are regularly confronted with patients who require immediate assistance but, to some extent, refuse treatment or cooperation [1]. In Denmark, the general principle of medical therapy is to respect the patient's autonomy; therefore, consent to treatment should always be obtained [2]. When an incapacitated patient, either permanently or due to psychosis or substance intoxication, is not competent to consent but refuses obviously required treatment, Danish legislation allows for exceptions from the consent requirement [2, 3, 4]. When consent cannot be obtained, one option is to persuade the patient; if that is not feasible, then one can turn to coercion in accordance with Danish legislation. In other cases, persuasion may be applied as an alternative to involuntary treatment. Emergency medical services (EMS) may be dispatched to patients who subsequently decline treatment [5, 6]. Accordingly, in some cases, consent cannot be obtained despite attempts to persuade the patient to consent, and coercion may be necessary to ensure treatment in life‐threatening conditions [1, 2, 3, 4]. This is an obvious breach of the patient's autonomy founded on a paternalistic approach to the patients.

In Denmark, each year, approximately 3000 adults are forcibly admitted to a hospital under coercion in accordance with the Danish psychiatry act due to psychosis or a mental state equating psychosis [7]. The existing literature on coercion has primarily focused on coercion within psychiatric wards, while little attention has been given to the use of coercion in the prehospital setting. The limited research on coercion in prehospital settings has focused mainly on the different types of coercion, patient characteristics, prehospital care providers' experiences, and the role of the police from a qualitative perspective [1, 8, 9, 10, 11, 12, 13, 14, 15]. Police involvement is common when patients resist treatment, as they are solely able to address options of moving citizens against their will in prehospital settings. This emphasises the collaborative role between EMS personnel and law enforcement [1, 15].

In Denmark, a distinction is made between patients who are considered psychotic, or in a mental state equivalent to psychosis, and patients who are temporarily or permanently unable to provide informed consent for other reasons when immediate treatment is required. Two separate legal frameworks govern these situations [2, 4].

In a prehospital setting, the use of coercive measures is limited to cases of acute need for treatment, in which healthcare providers prioritise the patient's well‐being over the patient's personal autonomy [16].

Ethical challenges may arise prehospitally when medical guidelines, legal requirements, and EMS personnel's professional values conflict, potentially resulting in moral distress [17]. Understanding the extent to which coercion is used in the prehospital setting is essential from both the patient's perspective and the prehospital care provider's perspective.

The aim of the study was to quantify the extent of prehospital coercion and to describe patient characteristics among adult patients exposed to coercion or persuasion in the first 6 months of 2022 in the Region of Southern Denmark.

## 2 Methods and Materials

### 2.1 Study Design

This is a descriptive study using prehospital medical record data from the Region of Southern Denmark from 1 January to 30 June 2022.

### 2.2 Defintions

The study investigates patient characteristics (sex, age, substance use and self‐harming behaviour), as well as the role of the police in cases where coercion or persuasion has taken place. The indication for either was that there was an acute need for treatment and that the patients were in a state where they were not able to give informed consent due to, for instance, psychosis or confusion due to substance abuse or organic disease.

The four categories were defined by the authors based on Danish legislation and regional ambulance guidelines. The purpose of the classification was to distinguish between situations involving a formal legal basis for coercion, that is, the application of the Danish psychiatry act and situations in which treatment or transport was achieved through persuasion of the patient. Cases of ‘coercion’ or ‘persuasion’ are categorised into four types: coercion (forced admission in accordance with legislation), necessity or an urgent need for treatment justifying the disregarding of patient autonomy, persuasion of the patient with police involvement, and persuasion of the patient without police involvement (see Table 1 for definitions). Persuasion is defined as a targeted, sometimes police‐assisted approach that emphasises encouraging patients to accept recommended treatment without the use of physical or judicial force. Routine clinical communication, recommendations or information about treatment options were not considered persuasion. In our study, persuasion was acknowledged only when the medical record described initial reluctance, refusal or absence of consent, and EMS personnel nevertheless succeeded in obtaining consent for treatment or transport without invoking a formal legal basis for coercion. In some cases, the patient agreed to enter the ambulance but did not explicitly agree to the subsequent transport to the hospital.

**TABLE 1:** Classification of types of coercion in treatment that breach the autonomy of prehospital patients.

Types of coercion	Regulation/legislation	Description of clinical practise
1. Coercion (forced admission)	The Psychiatry Act Section 5	The central point in the Psychiatry Act is that involuntary admission may only take place if the patient is psychotic or in a mental state equivalent to psychosis. It would be unjustifiable not to detain the person concerned for treatment [2]. In the Danish system, this assessment is documented through formal procedures commonly referred to as ‘red papers’ or ‘yellow papers’, indicating the legal basis for involuntary admission or compulsory examination, respectively. The police must be involved in all cases of involuntary admissions of psychotic patients, as it is the chief of the police who, by delegation to the police attorney, approves the involuntary admission and provides assistance with the admission [2].
2. Necessity or an urgent need for treatment	The Criminal Law Section 14 The Health Act Section 19	According to Section 14 of the Criminal Code, actions are exempt from punishment in emergencies when it is necessary to intervene for the benefit of the patient, healthcare professionals, or others [3]. Necessity is a principle used to prevent imminent harm to persons or property [3]. According to Section 19 of the Health Act, a healthcare professional may initiate or continue treatment without consent if a patient temporarily or permanently cannot give informed consent and immediate treatment is needed to ensure survival or significantly improve survival chances or treatment outcomes [4].
3. Persuasion with the involvement of the police	There is, in principle, no legal authority here, but under Section 31 of the Psychiatry Act, medical doctors are obliged to inform the patient of the considerations for using coercion.	An example of police persuasion is encouraging voluntary admission, either with police or EMS personnel present, and informing the patient that the police will be involved if the patient does not comply. It is explicitly explained to the patient that the patient will be forcibly admitted if he/she resist. When an indication for coercion exists, the Danish Psychiatry Act requires that the patient be informed accordingly, even though this information may, in some cases, lead to voluntary compliance. This is referred to as ‘information on planned use of coercion’ in the Danish Psychiatry Act [2].
4. Persuasion without the involvement of the police	There is no legal authority here.	An example of persuasion without the police is that voluntary hospitalisation is encouraged, but once the patient is placed on a stretcher or in the ambulance cabin, transportation to the hospital occurs with or without the patient's consent. Typically, this persuasion is used in patients obviously in need of immediate treatment. It usually involves vulnerable patients, such as those with delirium, who are not resisting treatment but may lack the capacity to provide informed consent [17]. This is an obvious brteach of the patient's autonomy funded in a paternalistic approach the patients and his/her needs.

*Note:* Coercion or persuasion is stratified into acts requiring the specific involvement of the police, the administration of treatment with legislative support and treatment administered after persuading the patient.

### 2.3 Setting

The Region of Southern Denmark has a population of 1,238,406 residents and covers 12,262 km^2^. The region is one of Denmark's five health regions responsible for regional healthcare. The healthcare system is publicly funded and free of charge to all citizens at the point of contact. Across all Danish health regions, the EMS consists of a three‐tiered system: ambulances staffed with two emergency medical technicians or paramedics, and rapid response vehicles staffed with either paramedics as a second tier or anaesthesiologists as the third tier [18]. The primary prehospital resource is an ambulance, which, in approximately 25% of cases, is supplemented by a rapid‐response vehicle [18]. All EMS personnel document treatments in a nationwide electronic prehospital medical record, which includes prehospital findings, vital parameters, administered treatments, and patient details (including the unique patient identifier, the Civil Personal Registration Number) [6, 19]. Documentation is completed during or immediately after treatment [6]. Free text can be added to describe incidents or observations. These notes also record any police involvement [6]. The data are instantly accessible to hospital emergency departments and psychiatric units, helping them prepare for incoming patients [20].

### 2.4 Sampling Procedure

We included all ambulance patients in Southern Denmark between 1 January 2022, and 30 June 2022. Patients without a valid Civil Personal Registration Number or aged younger than 18 years were excluded. In case of multiple contacts, each contact with the EMS was included.

### 2.5 Identification and Classification of Coercion

To identify cases of possible coercion, patients were initially screened electronically using registration data and text mining of free‐text fields in prehospital medical records [6, 20]. The relevant text and variables were identified and defined in collaboration with experienced prehospital physicians, psychiatrists, and emergency physicians.

In the initial screening, one or more of the following criteria had to be met:

1. A psychiatric diagnosis assigned to the patient by a prehospital anaesthesiologist present on scene.
2. Transport of the patient to a psychiatric department.
3. Free‐text listing of at least one of the following terms or derivatives thereof (translated from Danish): ‘police’, ‘coercion’, ‘force’, ‘restraint’, ‘distressed’, ‘psychosis’, ‘psychotic’, ‘red papers [Danish term for forcible admission to psychiatric ward]’, ‘externalizing behavior’, ‘violence’, ‘violent’, ‘insane’, ‘stand‐by’, ‘standby’, ‘prison’, ‘residence’, ‘handcuffs’, ‘pepper spray’, ‘suicidal’, ‘suicide’, ‘affect’, ‘self‐harm’, ‘persuasion’, ‘persuaded’, ‘psychiatric emergency department’, ‘aggressive’, ‘threat’, ‘weapon’, ‘insanity’.

Following this initial electronic screening, the first 100 cases were scrutinised by authors S.H.V. and S.M. to align the assessment criteria. Once agreement had been established, all included cases were manually scrutinised by the author S.H.V. alone. Cases where it was obvious that no coercion had been applied were excluded. Psychiatric patients where the free‐text in the medical record system explicitly stated that the patient was voluntarily transported to a psychiatric department were also excluded. All other cases were included in the analysis.

Expert consultation with an experienced prehospital physician (author S.M.) was obtained in all cases where the classification was unclear.

### 2.6 Variables

Coercion or persuasion was stratified according to the predefined criteria listed in Table 1. When multiple forms of coercion or persuasion were applied to a single patient, the most severe form was selected, as coercion is usually preceded by attempts to persuade the patient to enter treatment voluntarily.

Other variables were *sex* (male and female) and *age*, categorised into four groups (18–29, 30–49, 50–69 and 70+), and included as a continuous variable. *Substance use* was categorised into ‘Not under the influence of substances’ and ‘Under the influence of substances’. *Self‐harming behaviour* was stratified in three categories: (1) no self‐harming behaviour, (2) suicidal thoughts without actual self‐harm and self‐harm defined as non‐self‐threatening inflicting of physical injury on the patient and (3) suicide attempt. The degree of *police involvement* was categorised into two strata: with police involvement and without police involvement.

### 2.7 Statistical Methods

#### 2.7.1 Descriptive Statistics

Non‐parametric descriptive statistics are applied. Categorical variables are given as absolute numbers and percentages, while continuous variables are shown as medians with first and third quartiles.

Data management and analysis were conducted using Microsoft Excel 2019 (Microsoft, Redmond, Washington, USA) and SPSS (Armonk, New York, USA).

### 2.8 Ethical Considerations

This project was approved by the Judicial Office of the Region of Southern Denmark (Ref. No. 31–1521‐434), and no further approvals were required under Danish legislation [21]. Data were stored on an encrypted server. All data management was conducted in accordance with Danish and European regulations on the processing of personally identifiable data [22, 23].

## 3 Results

### 3.1 Categorising the Types of Coercion or Persuasion

**FIGURE 1:**
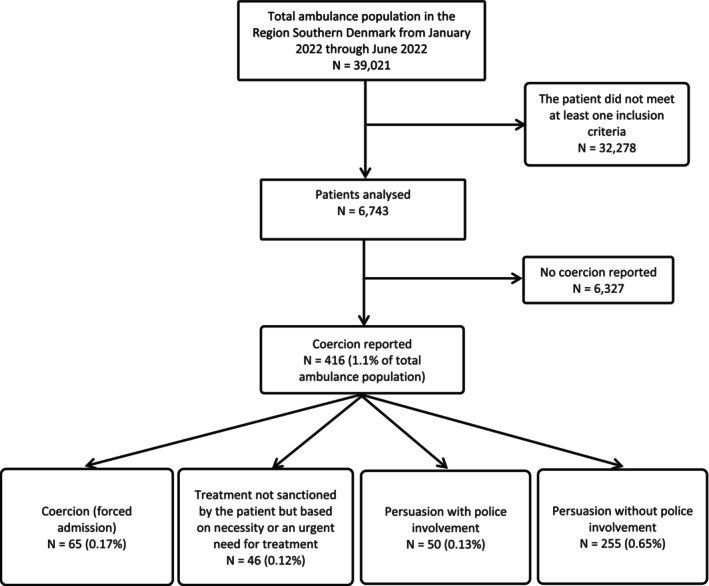
Flowchart of patient inclusion. Percentages reported in relation to the total ambulance population in the investigated period.

Among 39,021 prehospital missions during the study period, 6743 prehospital medical records met the screening criteria. The manual review revealed that 416 ambulance contacts (1.1% of all ambulance contacts) resulted in the use of coercion or persuasion. The distribution of different coercion types was: 0.2% of all ambulance contacts (65 cases) involved coercion as forced admission, in 0.1% of all ambulance contacts (46 cases), necessity or an urgent need for treatment resulted in treatment without the patient's consent. Of all ambulance contacts, 0.1% (50 cases) involved persuasion with police involvement, and 0.7% (255 cases) resulted in persuasion without police involvement. For Flowchart, see Figure 1.

### 3.2 Distribution Within the Study Population

Persuasion without police involvement was the most common form of coercion (61.3%). This was followed by coerced admission according to the Danish Psychiatry Act (15.6%), persuasion with police involvement (12.0%), and acts undertaken out of necessity or an urgent need for treatment (11.1%). Please refer to Table 2.

**TABLE 2:** Patient characteristics.

			Types of coercion							
	Total		1: Coercion (forced admission with a legal basis for involuntary admission or compulsory examination)		2: Necessity or an urgent need for treatment in emergencies when it is necessary to intervene for the benefit of the patient, healthcare professionals, or others		3: Persuasion with police, either with police or EMS personnel present, and informing the patient that the police will be involved if the patient does not comply.		4: Persuasion without police involvement applied to patients obviously in need of immediate treatment, usually involving vulnerable patients	
	*N*	%	*N*	%	*N*	%	*N*	%	*N*	%
Sex										
Male	228	54.8	33	50.8	27	58.7	26	52.0	142	55.7
Female	188	45.2	32	49.2	19	41.3	24	48.0	113	44.3
Age, median (quartiles)	56	(32/74)	33	(22/58)	42	(24/68)	45	(24/59)	66	(46/79)
Age categories										
18–29 years	89	21.4	26	40.0	17	37.0	17	34.0	29	11.4
30–49 years	83	20.0	12	18.5	13	28.3	14	28.0	44	17.3
50–69 years	103	24.8	16	24.6	6	13.0	12	24.0	69	27.1
70+ years	141	33.9	11	16.9	10	21.7	7	14.0	113	44.3
Substance abuse	112	26.9	14	21.5	15	32.6	25	50.0	58	22.7
Self‐harming behaviour or suicidal thoughts	30	7.2	10	15.4	5	10.9	5	10.0	10	3.9
Deliberate suicide attempt	44	10.6	13	20.0	8	17.4	11	22.0	12	4.7

*Note:* Sex and age characteristics among prehospital patients in whom coercion occurred. Incidence of substance abuse, self‐harming behaviour and suicidal thoughts and deliberate suicide attempts. Stratified by type of coercion or persuasion.

### 3.3 Population Characteristics

#### 3.3.1 Sex and Age

Most patients were male (54.8%). The median age of all patients was 56 years (Q1–Q3: 32–74 years). Males were over‐represented among patients exposed to coercion justified by necessity or an urgent need for treatment (58.7%), compared to the other types of coercion. Among the included patients, those aged 18–29 years more often had coerced admission (40.0%) and a recorded necessity or urgent need for treatment (37.0%). Patients aged 70 years or older were more frequently recorded as having persuasion without police involvement (44.3%) compared to younger age groups. For details, see Table 2.

#### 3.3.2 Substance Abuse

Overall, 112 (26.9%) patients exposed to any form of coercion were intoxicated. For details, see Table 2.

#### 3.3.3 Self‐Harming Behaviour

Among the 416 patients subjected to any form of coercion, 44 (10.6%) had attempted suicide, and 30 (7.2%) showed signs of self‐harm or expressed suicidal thoughts. Suicide attempt was reported among 22.0% of patients exposed to persuasion with police involvement and 20.0% of patients forcibly admitted to the hospital. For details, see Table 2.

#### 3.3.4 Police Involvement

In total, the police were involved in 131 (31.5%) of all the coerced patients. Sixty‐five of these patients were forcibly admitted to hospitals under the Danish Psychiatry Act. Sixteen of the patients who were subjected to treatment justified by necessity or an urgent need for treatment experienced some degree of police involvement, and 50 patients were persuaded with police involvement and brought to the hospital.

## 4 Discussion

In 1.1% of all prehospital adult patient encounters in the Region of Southern Denmark in the first half of 2022, a patient was coerced or persuaded into receiving necessary treatment without their consent. The most frequent action was persuasion without police involvement. In one patient in nine, EMS personnel considered the need for involuntary treatment not sanctioned by the patient to be justified because of an immediate threat to the patient, should the patient not be treated, or because it was related to the self‐defence of the EMS personnel. One patient in nine was exposed to persuasion with police involvement.

Prehospital coercion or persuasion has previously only been discussed to a limited extent in the literature [11, 15, 24]. One Scandinavian study suggests that 72% of Norwegian paramedics had, at some point in the past months, exerted some form of coercion toward patients [11, 15]. Although direct comparison with our study is limited because the Norwegian studies examined paramedics' views on coercion, whereas the present study examines what was actually recorded in medical charts, both studies indicate that coercion or persuasion is relatively frequent in prehospital settings. Differences in data sources and definitions of coercion likely contribute to some variation, as the Norwegian studies include actions that may not be systematically documented in the prehospital medical record [1, 15].

In our study, 61.3% of patients were persuaded without police involvement. One may speculate that factors such as time efficiency and ethical considerations may explain why EMS personnel avoid involving the police unless absolutely required [17]. Our study did not include or elaborate on the reasons for this approach, nor was it covered in the material. The rationale for ambulance personnel's use of persuasion and the effects of this breach of the patient's autonomy on personnel and patients requires further attention. It may be difficult to strike a balance between personal convictions about what constitutes good care and the patient's perceived dissonant care preferences [25]. Among the included patients, those aged 70 years or older were more frequently subjected to persuasion without police involvement. This may reflect a different clinical pattern in treating older patients, or a less direct objection from these patients. We can speculate that this finding reflects a mental or physical frailty that may reduce the patient's ability to resist transportation or treatment. Another potential explanation is that older patients may be more inclined to comply with authority, prompting EMS personnel to attempt to persuade patients who are initially unwilling to accept the treatments offered.

The ambulance guidelines in Denmark emphasise promoting voluntary cooperation before using coercion. This study's findings align with those of previous studies, which found that the least forceful method, persuasion without police involvement, an approach most commonly found in the material [26, 27]. However, the lack of police supervision might raise concerns about the inconsistent use of coercion or persuasion.

This study revealed that the police were involved in 31.5% of all coercion cases and, to some extent, also in prehospital situations involving intoxicated patients. This aligns with findings from a Norwegian study [1]. While police presence can ensure safety, it may be perceived as psychotraumatic for patients. The intricate dynamics between EMS personnel and the police emphasise the need for competence and knowledge within the four coercion or persuasion categories, ensuring police assist when necessary to prevent harm while minimising any potential psychological or physical distress to the patient.

On the other hand, a study on a Danish ambulance population found that one in five patients had a history of mental illness [28]. It has been previously shown that treating acutely ill patients against their consent can be mentally and ethically challenging for EMS personnel and may cause moral distress [17]. Such moral distress may arise when EMS personnel experience uncertainty or lack sufficient tools to adequately assess, interpret, and respond to patients' underlying needs, particularly in populations with a compromised ability to give informed consent. These results suggest a lack of understanding of patients' needs among EMS staff and suggest that further training in caring for vulnerable patients may be required [17, 28].

### 4.1 Strengths and Limitations

Using a large dataset from 6 months of prehospital medical records in a region covering 20% of Denmark's population improves internal validity. The systematic and thorough review of 6743 prehospital medical records, along with clear inclusion criteria and expert input, ensured consistent classification of variables and strengthened the study's internal validity. A limitation is that the manual review and classification of cases were primarily conducted by one researcher. Although unclear cases were discussed with an experienced prehospital physician, independent assessments by two researchers and a formal evaluation of inter‐rater agreement would have strengthened the reproducibility and reliability of the case identification and classification process.

The absence of predefined registration options for certain variables, such as police involvement, posed a risk of misclassification. We were limited to using data entered into the prehospital medical record system as free text rather than standardised registration fields. Basing studies on the sensitive issue of treating patients against their will on self‐reported entries in the medical records introduces a clear risk of under‐reporting. The risk of under‐reporting is likely to be greatest for less formal and less intrusive forms of persuasion, as these may not always be perceived as coercive by EMS personnel and may therefore be documented inconsistently. It is therefore possible that under‐reporting has occurred here, particularly when comparing with other studies reporting a much larger incidence of persuasion or coercion [11, 15]. Furthermore, case identification relied on free‐text documentation, and there is no standardised or mandatory registration of coercion or persuasion in the prehospital medical record. Consequently, some cases may not have been captured by the search strategy, particularly when police involvement or persuasion was not explicitly documented. There is also potential for recall bias and social desirability bias, as EMS personnel may underreport or inconsistently document coercion, particularly in less intrusive forms [29, 30]. As this study included only adult patients and was based on ambulance contacts from the first 6 months of 2022, the findings should be interpreted within this context.

We do, however, consider our findings generalisable within the Region of Southern Denmark; however, they may not fully apply to other regions or countries due to differences in laws, healthcare systems, and cultural practices.

## 5 Conclusion

The most frequently reported form of coercion is persuasion without any involvement of the police. The study highlights some clinically informative aspects, demonstrating that several forms of coercion or persuasion—even beyond those covered by psychiatric legislation—are applied in practice. This study identifies the frequency and types of coercion or persuasion used in the prehospital setting and highlights the clinical challenge of treating without consent.

## Author Contributions

Conception of the study, collection of data, writing of the first draft, approval of manuscript: Sif Højmark Vobbe. Defining and validation of search strategy, interpretation and analysis of data, defining any renewed search parameters, consultant in psychiatric context, revision of draft, approval of manuscript: Henriette Bruun. Conception of the study, defining search strategy, interpretation of data, revision of draft, approval of manuscript: Anne Craveiro Brøchner. Conception of study, defining search strategy, interpretation of data, revision of draft, approval of manuscript: Annmarie T. Lassen. Execution of search in the prehospital medical record system, further qualification of the search strategy in the electronic medical patient record system, revision of draft, approval of manuscript: Tim Alex Lindskou. Execution of search in the prehospital medical record system, further qualification of the search strategy in the electronic medical patient record system, revision of draft, approval of manuscript: Torben Anders Kløjborg. Consultant in psychiatric context, interpretation of data, revision of draft, approval of manuscript: Signe Wegmann Düring. Conception of the study, defining and validation of search strategy, collection of further data when necessary, interpretation of data, revision of draft, approval of manuscript: Søren Mikkelsen.

## Funding

Funding was acquired from the Region of Southern Denmark's Fund for Free and Strategic Research.

## Ethics Statement

This project was approved by the Judicial office of The Region of Southern Denmark (Ref. No. 31‐1521‐434), and no further approvals were required under Danish law. Data were stored on an encrypted server, and all data management was conducted in compliance with Danish and European regulations on personally identifiable data. Approval for storing and handling was obtained from the judicial office of Odense University Hospital (J. no. 24/4483). The data were pseudo‐anonymised, ensuring that individuals could not be identified. All data processing adhered to GDPR, and results were presented at the group level (with > 3 individuals per cell).

## Consent

The authors have nothing to report.

## Conflicts of Interest

The authors declare no conflicts of interest.

## Data Availability

According to Danish Legislation, no transfer of the dataset is allowed.
